# To a Zebra Finch: How the Brain Cultivates Birdsong

**DOI:** 10.1371/journal.pbio.0030162

**Published:** 2005-03-29

From the “ecstatic sound” of Thomas Hardy's thrush to the “full-throated ease” of Keats's nightingale, the dulcet tones of songbirds have long inspired poetic explorations of the human spirit. Scientists have more recently found inspiration in songbirds, but it is their behavior and not their song that tickles the scientific imagination. Just as the vocal explorations of toddlers reflect the (no doubt) consequential conversations of their elders, the highly variable chirps and warbles of juvenile songbirds echo the precise melodies of the adult songbird. Through trial and error and random forays into harmolodic dissonance, the young bird patterns his performance after a tutor song (usually performed by dad) until he produces a workable facsimile. It is this behavior— known as reinforcement learning—that makes songbirds an ideal model for studying the interplay between experience, brain activity, and learning.

Michale Fee's lab studies the neural basis of song learning in the zebra finch, the organism of choice for birdsong researchers. In a new study, Bence Ölveczky, Aaron Andalman, and Fee study just how young songbirds generate the vocal explorations that help the apprentice master its song.

Two major neural pathways control zebra finch song. The motor pathway controls vocal outputs through the RA (for robust nucleus of the arcopallium) neuron cluster, which indirectly stimulates vocal and respiratory muscles. When adult birds sing, RA neurons show a signature sequence of bursts during each syllable. Another pathway, called the anterior forebrain pathway (AFP), appears to be critical for song learning. AFP shares characteristics with the mammalian basal ganglia, which regulates movement and motor learning in mammals.[Fig pbio-0030162-g001]

**Figure pbio-0030162-g001:**
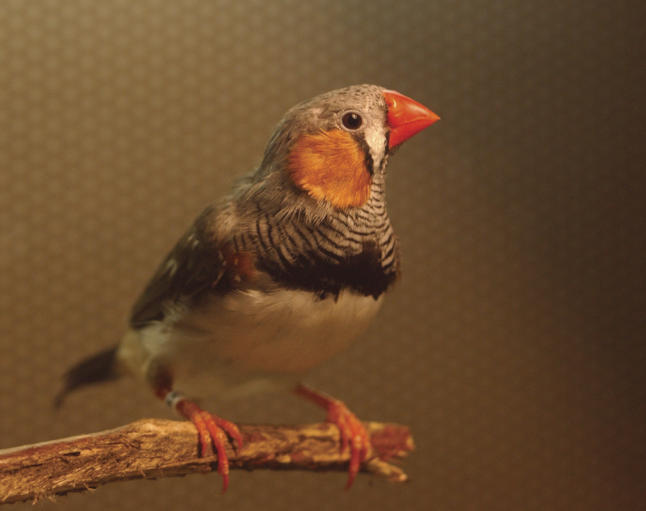
The vocal explorations of young zebra finches shed light on the neural basis of learning motor tasks (Photo: Daniel D. Baleckaitis)

To explore the nature of the AFP's contributions to song learning, Fee and colleagues recorded brain activity from young zebra finches (54–79 days old) learning to sing. Then they injected young birds with drugs that temporarily blocked activity in a brain region that is part of the AFP called LMAN (lateral magnocellular nucleus of the nidopallium). Zebra finch songs typically contain three to seven syllables—the basic acoustic units of zebra finch songs—that follow a specific sequence. Thirty to 90 minutes after LMAN inactivation, the birds sang with less syllabic variation. This effect was especially dramatic in the youngest birds, which normally exhibit the greatest acoustic variation. LMAN inactivation, the authors note, “eliminated 75% of the difference in mean variability between juvenile song and adult directed song [wooing a mate, for example]—the most stereotyped form of song.” LMAN inactivation also reduced the birds' variation in syllable sequence, which again hewed closer to the orthodoxy of adult song than to the exuberance of youthful experimentation.

The authors go on to show that changes in the firing patterns of LMAN neurons projecting into the motor pathway accompany changes in song. That LMAN inactivation reduces song variability quickly and reversibly, the authors argue, indicates that LMAN supports experimental behavior and controls song variability by providing rapid inputs to the motor pathway. This model requires that LMAN neurons show high variability across different song motifs—which is what Fee and colleagues found. As the bird sings, some as yet unknown brain areas must also evaluate the song against a template, modulating the actions of the motor pathway as a conductor might correct a performer's mistakes in note and pitch until she masters the tune.

It's thought that birdsong serves multiple purposes—staking a territorial claim, for example, and attracting a mate—though precisely how the song relates to fitness is still an open question. Whether inducing the type of exploratory motor behavior that's so critical to motor learning is a fundamental feature of basal ganglia circuits also remains to be determined. But it does seem clear that these circuits play a significant role in generating the variability that songbirds need in order to acquire the communication skills of their parents—a finding that may shed light on how the brain produces the fluctuations required for learning other tasks. For more on song learning, see the primer by Fernando Nottebohm (DOI: 10.1371/journal.pbio.0030164, available online May 2005).

